# Comparative metabolomics of acetylcholinesterase and α-glucosidase inhibitors in pericarp of *Garcinia mangostana* L.

**DOI:** 10.1186/s40529-025-00460-4

**Published:** 2025-05-21

**Authors:** Yun-Han Wang, Ta-Wei Liu, Sui-Wen Hsiao, Man-Hsiu Chu, Tzong-Huei Lee, Su-Jung Hsu, Shih Yin Chen, Ching-Kuo Lee

**Affiliations:** 1 Ph.D. Program in Clinical Drug Development of Herbal Medicine, Taipei Medical University, 250 Wu Xin Street, Taipei, 110 Taiwan; 2 School of Pharmacy, Taipei Medical University, 250 Wu Xin Street, Taipei, 110 Taiwan; 3 Ph.D. Program in Drug Discovery and Development Industry, Taipei Medical University, 250 Wu Xin Street, Taipei, 11031 Taiwan; 4 Institute of Fisheries Science, National Taiwan University, Taipei City, Taiwan; 5 Graduate Institute of Pharmacognosy, Taipei Medical University, 250 Wu Xin Street, Taipei, 11031 Taiwan; 6 School of Food Safety, Taipei Medical University, 250 Wu Xin Street, Taipei, 11031 Taiwan; 7 Department of Chemistry, Chung Yuan Christian University, 200 Zhongbei Road, Zhongli District, Taoyuan, 32023 Taiwan; 8 Xantho Biotechnology Co., Ltd. Rm ER15, 17F.−1, No. 3, Yuanqu St., Nangang Dist., Taipei City, 11503 Taiwan (R.O.C.)

**Keywords:** Mangosteen (*Garciniaman gostana* L), Acetylcholinesterase, α-glucosidase, Dereplication, Comparative metabolomics

## Abstract

**Background:**

Mangosteen (*Garcinia mangostana* L.) pericarp extract has demonstrated potential against Alzheimer’s disease (AD) and diabetes mellitus (DM). This study introduces a rapid dereplication and comparative approach to identify and characterize acetylcholinesterase (AChE) and α-glucosidase inhibitors in mangosteen pericarp. Using protein-subtraction, MS profiling, and computational modeling is effective for screening, identifying, and analyzing enzyme-inhibiting compounds from plant sources, and quantitative analysis of the main components has been performed.

**Results:**

The Mangosteen pericarp extract observed significant inhibitory activity against α-glucosidase and AChE, with IC50 values of 31.02 and 70.56 µg/mL, respectively. By comparing profiles of protein-subtracted extracts with non-treated extracts, eight potential inhibitors for each enzyme were identified: 8-desoxygartanin, gartanin, 3-isomangostin, β-mangostin, 9-hydroxycalabaxanthone, γ-mangostin, α-mangostin, and garcinone E. The α-mangostin was the most abundant, comprising 39.589% of the extract. Molecular docking revealed these inhibitors target the peripheral anionic site of AChE and the active site of α-glucosidase, forming key hydrogen bonds and pi-pi stacking interactions.

**Conclusion:**

This study emphasizes mangosteen pericarp as a promising natural source of these inhibitors, with potential for use in developing nutraceuticals and pharmaceuticals. The study validated a systems biology approach by applying dereplication and comparative UPLC-ESI-MS/MS metabolomics profiling to identify target-binding molecules in both protein-treated and untreated plant extracts. Further confirmation was obtained through molecular docking predictions, mechanism analysis, and compound quantification assays.

**Supplementary Information:**

The online version contains supplementary material available at 10.1186/s40529-025-00460-4.

## Background

Alzheimer’s disease (AD) is a widely recognized neurodegenerative disorder and has emerged as a significant public health concern in developed countries (Wang et al. 2012). In patients with AD, a deficiency in the neurotransmitter acetylcholine (ACh) has been observed in the brain. Inhibition of acetylcholinesterase (AChE), which breaks down ACh, is considered a primary therapeutic option for AD and helps to maintain the lifespan of neurotransmitters in the cerebral cortex (Phyu et al. 2014). Conversely, α-glucosidase is primarily utilized as a key enzyme in the treatment of metabolic disorders associated with diabetes mellitus (DM) and plays an essential role in carbohydrate metabolism by breaking down starch and disaccharides into glucose (Malik et al. 2024). Therefore issues with insulin secretion or insulin resistance in the body can result in elevated blood sugar levels and disrupted carbohydrate metabolism regulation, ultimately contributing to the development of diabetes (Mills et al. 2022), while the adverse effects of this medication may lead to complications in the cardiovascular and nervous systems and be associated with symptoms such as delayed wound healing, polyuria, and fatigue (Wang et al. 2022). Currently, oral medications approved for the treatment of hyperglycemia in type 2 diabetes including α-glucosidase inhibitors are an effective approach for the treatment of DM (Wang et al. 2022).

Mangosteen (*Garcinia mangostana* L.), often referred to as the “queen of fruits,” is a well-known tropical fruit that has a rich history of use as a medicinal plant (Aizat et al. 2019). However, a substantial amount of waste is generated during the processing of this fruit, as approximately 60% of the fruit consists of inedible rind, considered as by-products highlighted that mangosteen pericarp exhibits superior antioxidant activity and phenolic compound content compared to the aril, underscoring the high-value utilization of these by-products (Khaw et al. 2014, 2020a, b). In traditional medicine, mangosteen pericarp has been used to treat various conditions, including skin infections, diarrhea, and fever (Pedraza-Chaverri et al. 2008). Recent reports have discovered both fat-soluble substances (such as tocopherols and fatty acids) and water-soluble substances (including organic acids and phenolic compounds) from mangosteen pericarp extracts, which possess antioxidant, anti-inflammatory, anti-proliferative, and antibacterial properties (Albuquerque et al. 2023). Additionally, it has been reported that mangosteen pericarp extracts exhibit inhibitory effects on α-amylase, which could make them useful as functional supplements for blood sugar management and diabetes prevention, mainly due to their high polyphenol content (Li et al. 2022). Xanthones, a type of polyphenolic compound extracted from mangosteen pericarp, have been reported to exhibit biological effects in cardiovascular diseases, gastrointestinal diseases, osteoporosis, and cognitive impairments (Márquez-Valadez et al. 2009). In addition, xanthones have been found to have therapeutic potential in animal models of neurodegenerative diseases, reducing the progression of diseases such as Alzheimer’s disease, Parkinson’s disease and depression (Oberholzer et al. 2018; Do and Cho 2020). This suggests that xanthones could be a potential source of AChE inhibitors; however, there have been limited reports on the inhibitory components of AChE and α-glucosidase from mangosteen pericarp, and these components have not yet been identified, possibly due to a lack of effective methods.

Previously, we established a molecular interaction-based dereplication method, employing UPLC-ESI-MS/MS for comparative metabolomics analysis. Through the integration of molecular docking algorithms, we rapidly screened and identified xanthine oxidase inhibitors, elucidating their mechanisms of action (Hsu et al. 2021). Consequently, we posit that this approach holds promise for the study of other enzymes. The hypothesis was that this process primarily involved a comparative analysis of chromatograms between the enzyme-treated extracts and untreated extracts, the disappearance of peaks in the treated extracts suggests potential enzymatic oxidation or affinity for the enzyme, which was regarded as a promising enzyme inhibitory candidate. Conversely, peak increments could be linked to the presence of oxidation products.

Thus, the present study applied the previously proposed rapid dereplication system, differences between the metabolomic profiles of treated and untreated extracts measured using LC-ESI-MS/MS. ultimately identifying the compounds with acetylcholinesterase and α-glucosidase inhibitors from mangosteen pericarp. This facilitated an accelerated exploration of bioactive compounds within this botanical source. Our findings underscore not only the viability of this methodology but also its potential for expedited screening and identification of valuable compounds from natural resources, and we also report the quantitative results of the active substance xanthones.

## Methods

### Chemicals

All the experimental reagents, including *n*-hexane, ethyl acetate, ethanol, and butanol (Reagent grade), were purchased from Macron Fine Chemicals^TM^ (Radnor, PA, USA). Phosphate buffer (NaH2PO4), sodium phosphate dibasic (Na2HPO4), eserine (cat. 57-47-6), quercetin (cat. 6151-25-3), dimethyl sulfoxide (DMSO), acetylcholinesterase (EC 232-559-3) and α--glucosidase (EC 232-604-7) were purchased from Sigma Aldrich (St. Louis, MO, USA). MS grade methanol and acetonitrile were obtained from Merck (Darmstadt, Germany). The ultra-pure water was prepared with the Millipore-Q water purification system (Bedford, MA, USA). The standards including gartanin, garcinone E, α-mangostin, and γ-mangostin were purchased from Sigma Chemical Co (St. Louis, MO, USA). 8-desoxygartanin, 9-hydroxycalabaxanthone, 3-isomangostin, and β-mangostin were purchased from Wuhan ChemFaces Biochemical Co., Ltd. (Wuhan, Hubei, PRC).

### Preparation of pericarp of mangosteen extracts

Mangosteen (*Garcinia mangostana* L.), provided by Xantho Biotechnology in Taipei City, Taiwan. The pericarps of fresh mangosteen fruit were removed and dried. Then two kilograms of the pericarps was smashed and extracted three times with 95% ethanol at room temperature for one week, and obtained the crude extract 149.82 g.

### Isolation of components pericarp of mangosteen by medium-pressure liquid chromatography (MPLC)

After partitioning, 80 g of the ethyl acetate layer from the mangosteen pericarp was taken and coated onto 96 g of silica gel. The separation of the extracts was conducted using a medium-pressure liquid chromatography (MPLC) system (the Isolera^TM^ One Uppsala instrument manufactured by Biotage, Sweden). The step-gradient of purification method was used to obtain the 0, 10, 20, 40, 60 and 100 (%) fraction ethyl acetate with *n*-hexane to collect 54 fractions (Fr.1-Fr.54).

### Assessment of enzyme inhibitory of mangosteen pericarps crude extracts acetylcholinesterase (AChE) inhibitory activity assay

The AChE inhibition assay was performed with slight modifications based on the method described by Ellman et al. (1961). Prepare the buffer solution was 0.1 M at pH 8.0 of NaH2PO4 and Na2HPO4, and 3.0 M 5,5-dithio-bis-(2-nitrobenzoic acid) (DTNB) solution by dissolving in the prepared phosphate buffer solution, adjusting the pH to 8.0. The final concentration of 0.03 U/mL AChE and 0.015 M acetylthiocholine iodide (ATCI) was achieved by using Tris buffer solution. The 96-well plate was separated into four groups including blank control (BC), experimental control (EC), sample control (SC) and sample experiment (SE). BC was prepared by adding 100 µL of DTNB solution to 80 µL of Tris buffer and 20 µL of ATCI. EC was prepared by adding 100 µL of DTNB solution to 60 µL of Tris buffer, 20 µL of ATCI, and 20 µL of AChE. SC consisted of 100 µL of DTNB solution, 60 µL of Tris buffer, 20 µL of ATCI, and 20 µL of the sample solution. SE comprised 100 µL of DTNB solution, 40 µL of Tris buffer, 20 µL of ATCI, 20 µL of sample solution, and 20 µL of AChE. The SE was diluted from 500 µg to 62.5 µg. After preparation, the absorbance values were read at a wavelength of 405 nm and constant temperature of 37 °C using microplate reader.

The percentage of AChE activity inhibition was calculated. The calculation formula for:

$$\begin{aligned}&\text{A}\text{c}\text{h}\text{E}\:\left(\text{a}\text{c}\text{e}\text{t}\text{y}\text{l}\text{c}\text{h}\text{o}\text{l}\text{i}\text{n}\text{e}\text{s}\text{t}\text{e}\text{r}\text{a}\text{s}\text{e}\right)\:\text{i}\text{n}\text{h}\text{i}\text{b}\text{i}\text{t}\text{o}\text{r}\text{y}\:\left(\%\right)\cr&\quad=\left[\right(\text{E}\text{C}-\text{B}\text{C}\left)\right]/(\text{S}\text{E}-\text{S}\text{C})]\text{*}100\%\end{aligned}$$

SE = absorbance of sample experiment (with AChE).

SC = absorbance of sample control (without AChE).

EC = absorbance of experimental control (with AChE).

BC = absorbance of blank control (without AChE).

### α-Glucosidase inhibitory activity assay

α-Glucosidase inhibition activity of mangosteen pericarps crude extract and pure compounds was measured following the method described previously (Palanisamy et al. 2011). Prepare the buffer solution was 0.1 M at pH 6.8 of NaH2PO4 and Na2HPO4. A 4.0 mM *p*-nitrophenyl-α-D-glucopyranoside (*p*-NPG) substrate solution and 0.8 U/mL of α-glucosidase was prepared using a phosphate buffer solution. Furthermore, positive control group was prepared with 20 µg/mL of quercetin and the sample was initially prepared at a concentration of 125 µg and then diluted by half serial dilution to a final concentration of 7.8 µg. The 96-well plate was separated into four groups including blank control (BC), experimental control (EC), sample control (SC) and sample experiment (SE). For the BC, 83 µL of phosphate buffer solution was added to 17 µL of *p*-NPG. In the EC, 66 µL of phosphate buffer solution, 17 µL of *p*-NPG, and 17 µL of α-glucosidase were combined. The SC included 53 µL of phosphate buffer solution, 30 µL of sample solution, and 17 µL of *p*-NPG. The SE consisted of 36 µL of phosphate buffer solution, 30 µL of sample, 17 µL of *p*-NPG, and 17 µL of α-glucosidase. After the preparation was completed, the reactions were incubated at 37 °C for 5 min, followed by the addition of 100 µL of Na2CO3 to stop the reactions. Finally, a microplate reader was used to measure the absorbance values at a wavelength of 400 nm and a constant temperature of 37 °C. The calculation formula for α-glucosidase inhibition rate (%) is as follows:

$$\begin{aligned}&\upalpha-\text{g}\text{l}\text{u}\text{c}\text{o}\text{s}\text{i}\text{d}\text{a}\text{s}\text{e}\:\text{i}\text{n}\text{h}\text{i}\text{b}\text{i}\text{t}\text{o}\text{r}\text{y}\:\left(\text{\%}\right)\cr&\quad=\left[\right(\text{E}\text{C}-\text{B}\text{C}\left)\right]/(\text{S}\text{E}-\text{S}\text{C})]\text{*}100\text{\%}\end{aligned}$$

SE = absorbance of sample experiment (with α-glucosidase).

SC = absorbance of sample control (without α-glucosidase).

EC = absorbance of experimental control (with α-glucosidase).

BC = absorbance of blank control (without α-glucosidase).

### Preparation of enzyme-subtracted extract for UPLC-MS/MS analysis

#### UPLC-MS/MS analysis of acetylcholinesterase and α-glucosidase inhibitory

The method described by Hsu et al. was followed with modifications to subtract acetylcholinesterase and glucosidase binding components from the mangosteen pericarp crude extract which 30 mg was dissolved in 150 µL DMSO and mixed with 650 µL of buffer (Hsu et al. 2021). The mixture was divided into two part, one added with 100 µL 0.3 U/mL acetylcholinesterase or 5.6 U/mL α-glucosidase and 200 µL of buffer, another mixed with 300 µL of buffer. The mixture was stirred for 40 min in a rotator at 37 °C. Then 900 µL of acetonitrile was added to terminate the binding reaction and to precipitate proteins and other large molecules. The obtained mixture, i.e., the acetylcholinesterase-subtracted extract and α-glucosidase-subtracted extract, containing mangosteen pericarp crude extract at a final concentration of 10 mg/mL, was filtered through a 0.22 μm membrane prior to UPLC-ESI-MS/MS analysis.

#### UPLC-MS/MS metabolomics analysis

To perform composition profiling, 5 µL of the clarified sample solution was analyzed using a Q Exactive^TM^ Plus Hybrid Quadrupole Orbitrap Mass (Thermo Fisher Scientific Inc., USA). The Thermo Polar Premium C18 column (150*2.1 mm*2.6 μm) was used as the analytical column. The mobile phase A consisted of ddH2O with 0.1% formic acid, and the mobile phase B consisted of acetonitrile with 0.1% formic acid. The flow rate was set to 0.3 mL/min, the initial elution gradient was set to 50% mobile phase B from 0 to 4 min, and then increased to 70–85% mobile phase B from 4 to 5 min, 5 to 12 min, the elution gradient was set to 85–90% mobile phase B, followed by 90–100% mobile phase B from 12 to 15 min, and 100% mobile phase B from 15 to 18 min. The raw data from LC-MS was processed and automatically using Xcalibur software (version 2.2, Thermo Fisher Scientific,) with the negative ion mode. In the analysis process, the mass spectrometer performed high-resolution (resolving power, *r* = 70,000) full scan cycles (m/z 150–2000). The HESI souse was set at these parameters: Spray voltage of -3.5 KV; capillary temperature was set at 256 °C and souse heater temperature was maintained at 413 °C.

### Dereplication of acetylcholinesterase and α-glucosidase inhibitory of UPLC–ESI–MS/MS metabolome profiles

To identify variances in the metabolome profiles between the crude extract (Blank) and the α-glucosidase and acetylcholinesterase protein-subtracted extract (Test), the retention time, m/z, and peak intensity data from three independent UPLC–ESI–MS/MS analyses were exported as text files by Progenesis QI v2.1 (Nonlinear Dynamics, Waters Corporation, UK). The chromatographic peaks’ intensity ratio between the Test extract and the Blank extract was determined using the provided equation. Peaks exhibiting an intensity change exceeding 10% were identified as significant alterations resulting from the α-glucosidase and acetylcholinesterase protein subtraction, suggesting their potential interaction with α-glucosidase and acetylcholinesterase. The corresponding chemical formulas and structures of these peaks were subsequently identified using the ChemSpider, Reaxys database, and commercial standards.

$$\:\text{I}\text{n}\text{t}\text{e}\text{n}\text{s}\text{i}\text{t}\text{y}\:\text{r}\text{a}\text{t}\text{i}\text{o}\:\left(\text{\%}\right)=\left[\right(\text{I}0-\text{I}\text{T})/\text{I}0]\times\:100\text{\%}$$

I0: intensity of Blank (crude extract without interaction with α-glucosidase and acetylcholinesterase protein.

IT: intensity of Test (α-glucosidase and acetylcholinesterase protein-interacted crude extract).

### Quantitative analysis on eight main components of mangosteen pericarp

Mangosteen Pericarps concentrate (100 mg) was dissolved in a 50 mL volumetric flask, diluted with 70% methanol, and sonicated for 1 h. Subsequently, diluent was added to reach the volume mark, the mixture was thoroughly mixed, and then filtered through a 0.22 μm NY membrane. The filtrate was additionally diluted 1000-fold for analysis. Next, each standard added with 70% methanol to prepare standard solutions at a concentration of 1000 ppm. Dilute and mix the standard solutions obtain a mixed standard solution with the following concentrations for each standard: 8-desoxygartanin, 9-hydroxycalabaxanthone: 2, 5, 10, 20, 40 ppb; gartanin: 2, 5, 10, 20, 40, 60 ppb; 3-isomangostin: 0.2, 0.5, 1, 2, 4, 6, 10 ppb; α-mangostin: 100, 200, 400, 600, 1000 ppb; β-mangostin: 1, 2, 4, 6, 10 ppb; γ-mangostin: 2, 10, 25, 50, 100, 150 ppb; garcinone E: 0.5, 2.5, 6.25, 12.5, 25, 37.5 ppb. UPLC–ESI–MS/MS was employed for quantitative analysis, and the chromatography gradient and machine parameters were the same as those mentioned for metabolomics analysis.

$$\begin{aligned}&\text{W}\text{e}\text{i}\text{g}\text{h}\text{t}\:\text{c}\text{o}\text{n}\text{c}\text{e}\text{n}\text{t}\text{r}\text{a}\text{t}\text{i}\text{o}\text{n}\:(\text{\%o}\:\text{w}/\text{w})\cr&\quad=\left(\right(\text{A}-\text{b})/\text{a})\times\:50\times\:1000\times\:10^{-6}/100\times\:1000\text{\%o}\end{aligned}$$

a, b: the slope and intercept of the calibration curve.

A: the peak area of each compound.

### Statistical analysis

For the enzyme experiment, we used GraphPad Prism 8 (GraphPad Software, Inc., San Diego, CA, USA) for multiple comparisons and performed statistical analysis of the data using one-way analysis of variance (ANOVA). All experiments were performed in triplicate, and the mean ± standard deviation was calculated for final statistical analysis. Differences were considered statistically significant when *p* < 0.05.

### Molecular Docking analysis for acetylcholinesterase inhibitors and α-glucosidase inhibitors

The CDOCKER algorithm in Discovery Studio 2021 (Accelrys Software, Inc., San Diego, CA, USA) was used to assess the probable molecular binding mode between eight potential AChE inhibitors and α-glucosidase inhibitors. Enserin and quercetin as positive control for the acetylcholinesterase and α-glucosidase, respectively. The Protein Data Bank (http://www.rcsb.org/pdb/) was used to obtain the crystal structure of acetylcholinesterase (PDB ID: 1C2B) and α-glucosidase (PDB ID: 3A4A). Compound structures were generated in ChemBioDraw Ultra 13.3, then converted to 3D formats and energy-minimized in DS 2021, then subjected to energy reduction in the CHARMm force field using the conjugate gradient approach (convergence criterion of 0.001 kcal/mol) (Brooks et al. 1983). Following that, by eliminating water molecules and adding hydrogen atoms, the co-crystallized structure of the acetylcholinesterase receptor (PDB code: 1C2B) and the acetylcholinesterase receptor (PDB code: 3A4A) were created. The available module was used to represent missing loop sections in the prepared protein. Subsequently, the protein structure was subjected to ionization and protonation computations, followed by a final round of energy minimization optimized for molecular docking.

The docking analysis binding site was determined by referring to the PDB site records and then updated using the binding site module. Upon which, the molecular docking analysis focused on the discovered binding site sphere within the 1C2B structure (coordinates: x = 34.409, y = 86.3318, z = 27.5385; radius = 21.474) and the 3A4A structure (coordinates: x = 14.8348, y=-13.4189, z = 16.5495; radius = 23.4). Using the CDOCKER program included in DS 2021, the energy-minimized structures of the eight selected candidate compounds, positive control, were docked into the binding site of 1C2B and 3A4A, respectively. The pose with the highest CDOCKER energy score was chosen as the best match for each chemical. Furthermore, for each complex, the ligand-binding free energy was calculated using the Generalized Born with Molecular Volume (GBMV) approach (Lee et al. 2003).

## Results

### Evaluation Inhibition of α-glucosidase and acetylcholinesterase activity from the crude extract of the mangosteen pericarps

**Fig. 1:**
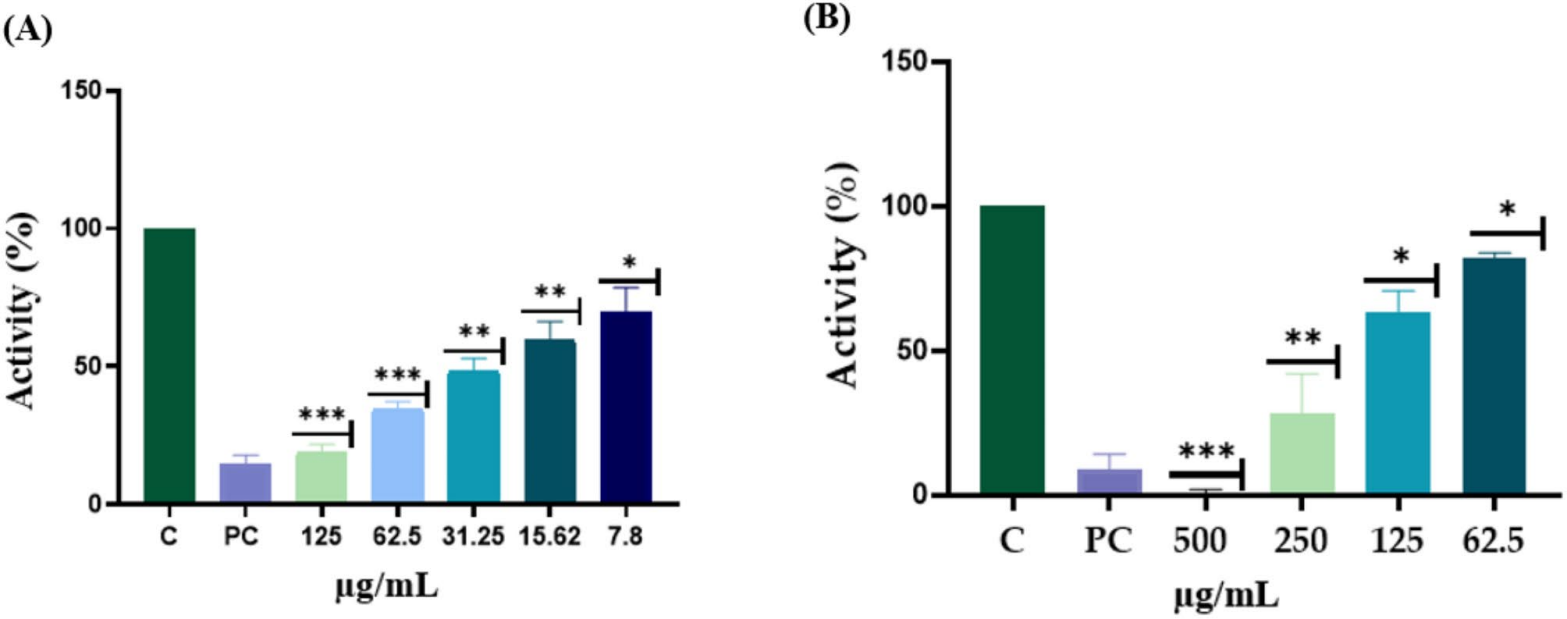
The comparative ratio of α-glucosidase and acetylcholinesterase inhibitory of the mangosteen pericarps. (**A**) α-Glucosidase inhibitory, positive control using quercetin 66 µM/mL. (**B**) acetylcholinesterase inhibitory, positive control using enserin 10µM/mL. * p-value < 0.05, **p-value < 0.01, *** p-value < 0.001 compared with control group

Previous studies have shown that the pericarp of the mangosteen discovered a rich content of polyphenolic compounds and xanthones which possess antioxidant activity, anti-inflammatory activity, anti-adipogenic activity and anti-α-amylase (Albuquerque et al. 2023; Li et al. 2022). In light of this, our initial approach involved subjecting the pericarp extract to in vitro analysis to determine its inhibitory effects against α-glucosidase and acetylcholinesterase. Results indicated that the crude extract of the mangosteen pericarp inhibitor activity against α-glucosidase, with its inhibitory activity showing a proportional increase in response to the concentration of the sample. Notably, at an escalated concentration of the crude extract, reaching 125 µg/mL, the inhibition rate nearly paralleled that of the positive control (quercetin), achieving an approximate 88% inhibition rate, and exhibiting50% inhibitory activity at 31.02 µg/mL (Fig. 1A). These findings corroborate prior research suggesting the existence of active compounds within the mangosteen pericarp that potentially inhibit α-glucosidase. Furthermore, to confirm the presence of potential neuroprotective compounds in the mangosteen pericarp, we conducted in vitro assays on AChE activity using the pericarp extract. As depicted in Fig. 1B, the inhibitory activity of AChE increased with an elevation in the concentration of the crude extract, with the inhibitory activity against AChE dropping below 50% at a concentration of 70.56 µg/mL (Fig. 1B). These results indicate the existence of neuroprotective compounds within the mangosteen pericarp.

### Identification and structure determination of potential α-glucosidase and acetylcholinesterase inhibiting constituents of mangosteen pericarps extract by enzyme-interacting dereplication of UPLC- MS/MS profiling

**Fig. 2:**
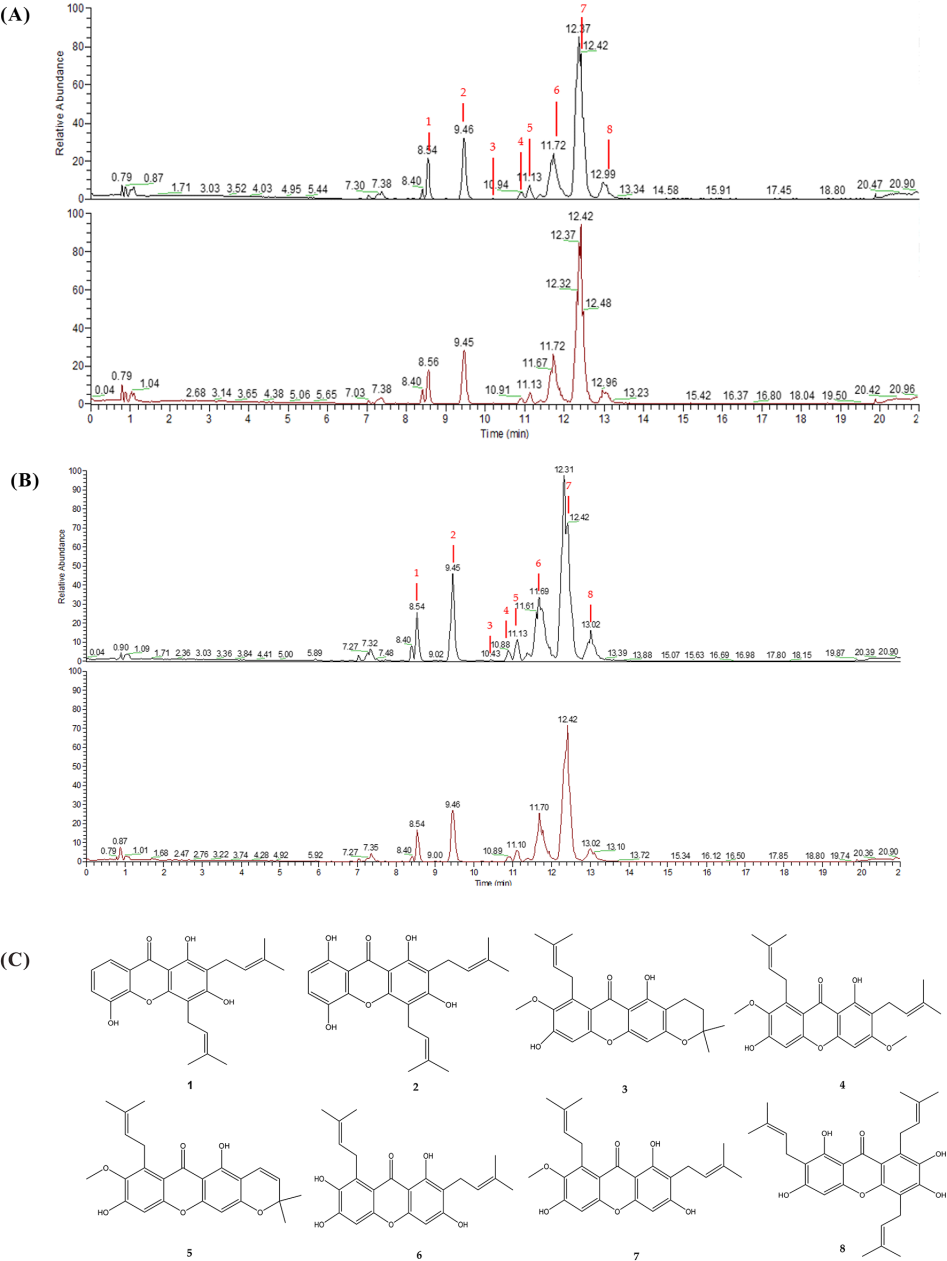
A comparative UPLC-ESI-MS profile of *Garcinia mangostana* L. pericarp crude extracts, with and without acetylcholinesterase and α-glucosidase subtraction, respectively, was conducted using negative ion mode. Eight peaks showing significant differences between the two extracts were highlighted by the numbered labels above. (**A**) Acetylcholinesterase. (**B**) α-Glucosidase. Black: without enzyme. Red: with enzyme. (**C**) The structures of eight potential α-glucosidase and acetylcholinesterase inhibitors were identified from mangosteen pericarp extract

To identify potential α-glucosidase and acetylcholinesterase inhibitors in mangosteen pericarps, the crude extract was initially incubated with α-glucosidase and acetylcholinesterase proteins. This allowed the extract components to interact with the enzymes. Compounds that bound to or were oxidized by the enzymes were potentially removed, resulting in the subtracted extract. The compositions of the crude extract (Blank) and the enzyme-subtracted extract (Test) were then analyzed using UPLC-ESI-MS in both positive and negative scan modes within the m/z range of 150–2000. Superior quality negative ion signals provided clearer chromatograms, facilitating data acquisition from the detected peaks. Representative BPC chromatograms are shown in Fig. 2A and B.

The UPLC-ESI-MS/MS metabolomic profiling of the mangosteen pericarp crude extract revealed numerous peaks. To identify potential compounds interacting with α-glucosidase and acetylcholinesterase, we compared the profiles of the crude extract and the enzyme-subtracted extracts. Peaks with an intensity decrease of more than 10% (Table 1) were deemed significant changes due to enzyme interaction. By calculating intensity changes and comparing base peak intensity in the chromatograms, eight peaks in Fig. 2A and B showed significant differences, indicating potential interaction with the enzymes. This dereplication method efficiently identified eight target compounds, demonstrating its advantage over conventional fraction-by-fraction analysis.

**Table 1:** Identification of the acetylcholinesterase inhibitors and α-Glucosidase inhibitors from pericarp of *Garcinia mangistana L.* by UPLC-MS/MS in negative ion mode

NO	Rt (min)	Measure m/z [M-H]^-^	Theoretical Formula [M-H]^-^	Error (ppm)	MS-MS fragments (m/z)	Identification	Reference
1	8.62	379.15460	C23H23O5	1.5	335.0923, 321.0758	8-desoxygartanin	(Liang et al. 2020)
2	9.46	395.14923	C23H23O6	0.7	351.0858, 297.0395	gartanin	(Li et al. 2016)
3	10.45	409.16547	C24H25O6	2.2	355.2857, 337.1511, 299.1862	3-isomangostin	(Khaw et al. 2020a, b)
4	10.91	423.18076	C25H27O6	1.2	408.1517,391.1971, 365.1016,353.2692, 307.0950,295.11703	β-mangostin	(Liang et al. 2020)
5	11.13	407.14969	C24H23O6	1.9	391.1976, 375.1659, 349.0679, 337.0706, 309.1738	9-hydroxycalabaxanthone	(Liang et al. 2020)
6	11.75	395.14969	C23H23O6	1.9	351.1666, 339.2001, 297.1346, 283.1188, 271.11880	γ-mangostin	(Liang et al. 2020)
7	12.42	409.16528	C24H25O6	1.7	394.1362, 377.1816, 351.0853, 339.2001, 311.1686, 283.1185	α-mangostin	(Liang et al. 2020)
8	13.02	463.21249	C28H31O6	2.1	419.1925, 407.1924, 365.1817, 351.0760, 339.2000, 283.1188	Garcinone E	(Liang et al. 2020)

### Structure identification of acetylcholinesterase and α-glucosidase-interacting components of the potential from the mangosteen pericarp

The eight potential α-glucosidase and acetylcholinesterase-interacting components were identified depending on the retention times, molecular weight and mass fragmentation pattern using the Progensis QI, then further demonstrated by comparing with those of the commercial standards (8-desoxygartanin and 9-hydroxycalabaxanthone, 3-isomangostin, Gartanin, Garcinone E, γ-mangostin, and α-mangostin). The identified compounds are listed in Table 1, and their structures are shown in Fig. 2C Xanthones, which are the most abundant components in mangosteen pericarp, are among these identified compounds (Khaw et al. 2014).

Eight significant peaks were identified, as follows: Peak 1 (Rt 8.62, [M-H]^-^ m/z 379.15460) corresponds to 8-desoxygartanin (Liang et al. 2020). Peak 2 (Rt 9.46, [M-H]^-^ m/z 395.14923) corresponds to gartanin (Li et al. 2022). Peak 3 (Rt 10.45, [M-H]^-^ m/z 409.16547) corresponds to 3-isomangostin (Khaw et al. 2014). Peak 4 (Rt 10.91, [M-H]^-^ m/z 423.18076) corresponds to β-mangostin (Liang et al. 2020). Peak 5 (Rt 11.13, [M-H]^-^ m/z 407.14969) corresponds to 9-hydroxycalabaxanthone (Liang et al. 2020). Peak 6 (Rt 11.75, [M-H]^-^ m/z 395.14969) corresponds to γ-mangostin (Liang et al. 2020). Peak 7 (Rt 12.42, [M-H]^-^ m/z 409.16528) corresponds to α-mangostin (Liang et al. 2020). Lastly, Peak 8 (Rt 13.02, [M-H]^-^ m/z 463.21249) corresponds to garcinone E (Liang et al. 2020).

### Quantification of eight ingredients in mangosteen pericarp

This study confirms that previously we developed a dereplication and comparative approach, potentially valuable compounds with affinity to the target enzymes could be rapidly identified by comparing the UPLC metabolomic profiles between enzyme-treated extracts and untreated extract materials (Hsu et al. 2021). In addition, we further quantified its content and showed that α-mangostin accounted for 39.589% of the total content, followed by 4.993% γ-mangosteen (Table 2). In addition, gartanin and garcinone E were also found to account for 1.603% and 1.562% of the total content, respectively.

**Table 2:** Quantification of eight ingredients in mangosteen pericarp

NO.	Compounds	Content in mangosteen pericarp Average ± SE(%)
1	8-desoxygartanin	1.005 ± 0.073
2	gartanin	1.603 ± 0.092
3	3-isomangostin	0.103 ± 0.008
4	β-mangostin	0.299 ± 0.015
5	9-hydroxycalabaxanthone	1.363 ± 0.083
6	γ-mangostin	4.993 ± 0.157
7	α-mangostin	39.589 ± 2.450
8	garcinone E	1.562 ± 0.018

### Mechanisms of the binding potency of eight ache inhibitors and α-glucosidase inhibitors

To further understand the binding affinity and mechanism of these eight active compounds in simultaneously inhibiting AChE activity and α-glucosidase activity by molecular docking. For comparison, quercetin and enserin were used as reference standards for AChE activity and α-glucosidase activity, respectively. Simulation calculations indicate that the higher the negative value of the CDOCKER interaction energy, the greater the binding affinity between the target protein and the inhibitor, resulting in a stronger interaction effect.

### Mechanisms of the binding potency of eight ache inhibitors

The active binding site of AChE has been extensively reported. Hydrolysis occurs near the bottom of the active site gorge, where forms a deep and narrow channel gorge. This channel is primarily composed of several distinct domains: catalytic, anionic, acyl, oxygen anionic and peripheral anionic site (PAS) (Dvir et al. 2010). The peripheral anionic site and catalytic anionic site are the main active sites of AChE. The peripheral anionic site acts as a transitional binding point for substrates, containing numerous aromatic amino acid residues as well as aspartic acid residues that can interact with the cationic substrate and guide them along the gorge toward the catalytic site (Dvir et al. 2010).

**Fig. 3:**
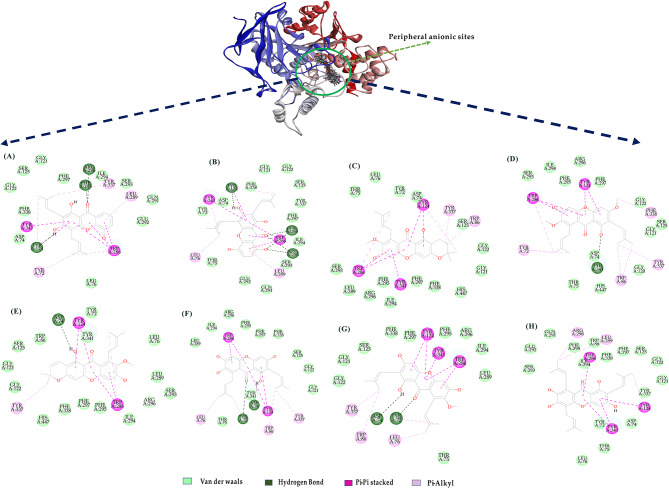
Binding orientations and interactions of the eight potential AChE inhibitors in the mangosteen pericarps to acetylcholinesterase (PDB code: 1C2B). View of docking 2D for the inhibitors binding to the peripheral anionic sites of acetylcholinesterase protein, (**A**) 8-desoxygartanin, (**B**) Gartanin, (**C**) 3-isomangostin, (**D**) β-mangostin, (**E**) 9-hydroxycalabaxanthone, (**F**) γ-mangostin, (**G**) ᾳ-mangostin, (**H**) Garcinone

Assessment of the binding affinity values of the eight potential AChE inhibitors (Table S1) showed that their binding energies ranged from 17.3201 kcal/mol to 27.0426 kcal/mol, comparable to that of enserin (as positive control). The docking conformation (Fig. 3) indicated that these inhibitors mainly accessed the peripheral anionic binding site of AChE to change the active site conformation. The peripheral anionic site is surrounded by aromatic amino acid residues (Trp286, Trp86, Tyr72, Tyr124, Tyr341, Tyr337, and Phe338) and the aspartic acid residue (Asp74), forming hydrogen bonds and hydrophobic interactions with the inhibitors. This observation aligns with the previously reported active site of ACE inhibitors (Dvir et al. 2010).

Furthermore, the docking conformations (Fig. 3A-C) demonstrate that α- mangostin, β-mangostin, and γ-mangostin share similar structures and exhibit comparable binding affinities toward AChE. Among them, α-mangostin displays higher affinity (-21.5838 kcal/mol). Its binding characteristics include hydrogen bonding with Asp74 and Tyr72, as well as pi-pi stacking interactions with aromatic amino acids (Trp286, Tyr124, and Tyr341). Additionally, it forms pi-alkyl hydrophobic interactions with Tyr337, Trp286, Tyr72, Trp86, and Phe338, indicating its ability to modulate the surface activity of the AChE peripheral site. Conversely, 3-isomangostin and 9-hydroxycalabaxanthone exhibit lower binding energies (17.3201 kcal/mol and 14.0434 kcal/mol, respectively), possibly due to hydrogen bonding with critical amino acids (Fig. 3). Notably, garcinone E demonstrates significant binding affinity to AChE, likely because it docks within the catalytic pocket, exerting inhibitory effects through hydrophobic interactions with Trp286, Tyr341, Tyr124, Leu289, and Arg296.

### Mechanisms of the binding potency of eight α-glucosidase

However, we conducted molecular docking studies on eight potential α-glucosidase inhibitors to investigate their interaction modes with the active site of α-glucosidase from Saccharomyces cerevisiae (PDB ID: 3A4A), which shares 84% similarity with human α-glucosidase. The selected inhibitors exhibited comparable docking scores and binding affinity values (Table S1). The results indicate that certain functional groups on the scaffolds of these eight xanthone compounds exhibit similar affinities in their interactions with α-glucosidase, with binding energy values ranging approximately from − 17.201 to -29.54 kcal/mol, all lower than Quercetin (as a positive control). The inhibition of α-glucosidase activity by these compounds is attributed to their high affinity for hydrogen bonds and hydrophobic interactions present in the narrow channel leading from the active site to the catalytic site.

**Fig. 4:**
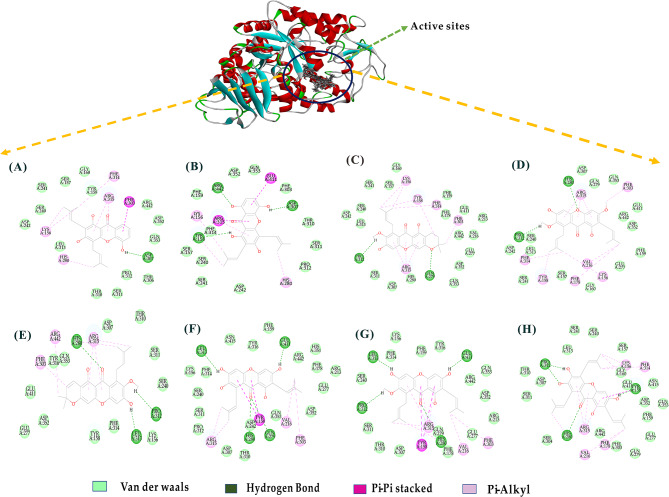
Binding orientations and interactions of the eight potential α-glucosidase inhibitors in the mangosteen pericarps to α-glucosidase (PDB code: 3A4A). View of docking 2D for the inhibitors binding to the active sites of α-glucosidase protein, (**A**) 8-desoxygartanin, (**B**) Gartanin, (**C**) 3-isomangostin, (**D**) β-mangostin, (**E**) 9 hydroxycalabaxanthone, (**F**) γ-mangostin, (**G**) ᾳ-mangostin, (**H**) Garcinone E

Docking conformations (Fig. 4A-C) suggest that α-mangostin, β-mangostin, and γ-mangostin have structurally similar compounds, but binding scores are influenced by hydrogen bonds and hydrophobic interactions between molecules and enzymes. Among them, α-mangostin exhibited the lowest binding energy of -29.54 kcal/mol, indicating high affinity. This is due to hydrogen bonding interactions formed with the key amino acids (Leu313, Pro312, Glu411, His280, Gln279) of the α-glucosidase protein. Additionally, α-mangostin and γ-mangostin form pi-pi stacking interactions with Tyr158 of the enzyme, while β-mangostin does not show it. All three compounds form hydrophobic bonds with Arg315, His280, Val216, and Phe303.

Although 3-isomangostin and 9-hydroxycalabaxanthone have similar structures, 3-isomangostin displays superior affinity due to hydrogen bonding interactions with Pro312 and Gln279 of the target enzyme, as well as critical hydrophobic interactions with amino acids (Arg315, Phe303, Phe314, Tyr158, Lys156) and surrounding van der Waals interactions. Similarly, 8-desoxygartanin and gartanin also have similar structures, with very similar binding energies, but gartanin exhibits better binding affinity at -19.0203 kcal/mol, consistent with trends reported in previous studies on α-glucosidase activity tests. This may be attributed to differences in important amino acids involved in hydrogen bonding and pi-pi interactions (Fig. 4). Furthermore, garcinone E also reveals a high affinity for the enzyme, with a value of -22.5433 kcal/mol, primarily due to the formation of four hydrogen bonds (Leu 313, Pro 312, Tyr158, and His 280) and pi-pi stacking interactions with Tyr158. Additionally, it forms hydrophobic interactions with Arg315, Phe178, Phe314, Tyr158, Lys156, Val216, and His280. In conclusion, the pi-pi stacking effect and hydrogen bond significantly contribute to the inhibitory activity of these inhibitors, which alter the conformation of the target enzyme by binding to the allosteric site, thereby reducing the substrate affinity for the active site.

## Discussions

In our study, α-mangostin was found to constitute 39.589% of the total content in the mangosteen pericarp extract, followed by γ-mangostin. This result is consistent with previous reports (Gondokesumo et al. 2019). Furthermore, gartanin and garcinone E were also detected in the mangosteen pericarp extract. Gondokesumo et al. found that the higher the maturity of mangosteen pericarps, the higher the garcinone E content (Gondokesumo et al. 2019). Therefore, these waste mangosteen by-products are worthy of development (Maulana et al. 2022).

α-Glucosidase is crucial in starch digestion and a key target in managing type 2 diabetes, as it converts complex carbohydrates into simple sugars. Located at the brush border of the small intestine, α-glucosidase hydrolyzes terminal (1→4)-linked α-glucosidase residues in starch and disaccharides, producing glucose. This process can slow carbohydrate digestion and reduce glucose absorption rates (Cai et al. 2017). Previous studies have shown that the ethanolic extract of *G. mangostana* pericarp exhibits hypoglycemic and antidiabetic activities, possibly by increasing insulin-producing β-cells in rats (Taher et al. 2016). However, previous studies have not shown that the components in mangosteen pericarp extract have the inhibitory effect on α-glucosidase. In our study, these eight compounds were shown to inhibit α-glucosidase activity through in vivo or in vitro tests. Research indicates that compounds from Garcinia mangostana, such as α-mangostin, β-mangostin, γ-mangostin, 8-desoxygartanin, 9-hydroxycalabaxanthone, and gartanin, effectively inhibit α-glucosidase with IC₅₀ values ranging from 1.5 to 34.2 µM, with γ-mangostin being the most effective mixed-type inhibitor. Additionally, α-mangostin has demonstrated potent antihyperglycemic activity in hyperglycemic rats (Ryu et al. 2011). A computer-based simulation study by Maulana et al. highlighted the potential of 3-isomangostin, α-mangostin, β-mangostin, and γ-mangostin as α-glucosidase inhibitors for anti-diabetic treatment (Maulana et al. 2022). Das et al. found that oral administration of dried ethanolic extract of α-mangostin (70.2 µg/mL) significantly inhibited α-glucosidase in Wistar rats (Das et al. 2019).

Not only that, Cardozo-Muñoz et al. found that *G. mangostana* fruits target α-amylase, α-glucosidase, and pancreatic lipase, key enzymes in carbohydrate and lipid metabolism, making them potential treatments for type 2 diabetes and obesity (Hu et al. 2021; Cardozo-Muñoz et al. 2022). In animal tests, Kunming mice given oral mangosteen ethanol extract (1.0 g/kg) showed inhibition of the diabetes drug target enzyme protein tyrosine phosphatase 1B (PTP1B), with garcinone E being the most potent (IC50 = 0.43 ± 0.11µmol/L). Other compounds, including 9-hydroxycucurbitanthinone (IC50 = 12.89 ± 1.53 µmol/L), 8-desoxygartanin (IC50 = 1.57 ± 0.29 µmol/L), γ-mangostin (IC50 = 0.86 ± 0.23 µmol/L), and α-mangostin (IC50 = 1.34 ± 0.34 µmol/L), also showed significant inhibitory effects (Thi et al. 2021). Additionally, Dvir et al. reported that garcinone E not only inhibited α-glucosidase (IC50 500 = 11.5 µM) but also exhibited cytotoxicity against four cancer cell lines.

However, our docking results for these eight compounds showed that 3-isomangostin and 9-hydroxycalabaxanthone have similar structures. 3-Isomangostin exhibited excellent affinity due to hydrogen bonding interactions with Pro312 and Gln279 of the target enzyme, as well as critical hydrophobic interactions with amino acids (Arg315, Phe303, Phe314, Tyr158, Lys156). Similarly, 8-deoxygartanin and gartanin also have comparable structures and display very similar binding energies, with gartanin showing better binding affinity at -19.0203 kcal/mol, consistent with previous reports on α-glucosidase activity tests. This may be attributed to differences in key amino acids involved in hydrogen bonding and π-π interactions. Overall, π-π stacking effects and hydrogen bonds significantly contribute to the inhibitory activity of these inhibitors, as they alter the target enzyme’s conformation by binding to the allosteric site, thereby reducing substrate affinity at the active site. Such inhibitors are considered mixed-type inhibitors.

On the other hand, in alignment with our study, Phyu et al. conducted in vitro experiments with xanthone aqueous extract from mangosteen, showing that it restored AChE activity in lead-exposed groups (Phyu et al. 2014). In vivo experiments also demonstrated the protective effects of this extract on AChE activity in red blood cells affected by lead (Phyu et al. 2014). Weecharangsan et al. examined the antioxidant and neuroprotective properties of four extracts (water, 50% ethanol, 95% ethanol, and ethyl acetate) from mangosteen pericarps (Weecharangsan et al. 2006). Water and 50% ethanol extracts showed high antioxidant activity (IC50 = 34.98 ± 2.24 and 30.76 ± 1.66 µg/mL, respectively) and demonstrated neuroprotective effects at 50 µg/mL. The 50% ethanol extract exhibited superior neuroprotective activity against NG108-15 cell line compared to the water extract (Weecharangsan et al. 2006).

Interestingly, although α-mangostin, β-mangostin, and γ-mangostin share a common chemical backbone, their differing numbers of hydroxyl and methoxy groups lead to slight variations in neuroprotective activities (Fig. 3). These findings suggest that the interaction between xanthones and the peripheral site may lead to conformational changes, spatial hindrance, or occupation of the surrounding space near the catalytic site within the enzyme, potentially resulting in decreased catalytic activity (Cardozo-Muñoz et al. 2022). Lee et al. found that γ-mangostin is more effective than α-mangostin in combating oxidative stress-related neurodegenerative diseases, including AD (Lee et al. 2019). Additionally, oral administration of γ-mangostin at doses of 10 and 30 mg/kg significantly improved scopolamine-induced memory impairment in mice (Lee et al. 2019). While β-mangostin has fewer reports on its effects on AChE, recent research by Le et al. confirmed its AChE inhibitory efficacy with an IC50 of 2.17 µM, consistent with our findings (Le et al. 2023) Oetari et al. reported that gartanin, the second-most abundant compound in mangosteen pericarps after α-mangostin, exhibits neuroprotective properties by clearing DPPH free radicals induced by glutamate in HT22 cell death (Oetari et al. 2019). Both gartanin and γ-mangostin can penetrate the blood-brain barrier in vitro (Wang et al. 2016). Gao et al. demonstrated neuroprotective ability of gartanin against glutamate-induced damage in HT22 cells via oxidative damage mechanisms involving the Nrf-2-independent HO-1 and the AMPK/SIRT1/PGC-1α pathways (Gao et al. 2016). Ryu et al. identified γ-mangostin, 8-desoxygartanin, and α-mangostin from mangosteen seeds as potent AChE inhibitors with IC50 values between 6.2 and 15.0 µM, also showing significant Butyrylcholinesterase (BChE) inhibitory effects with IC50 values from 0.7 to 11.0 µM (Ryu et al. 2014). BChE is linked to neurodegenerative diseases as it hydrolyzes various choline esters. Khaw et al. studied xanthones from mangosteen pericarp extract, including α-mangostin (IC50 = 2.14 µM), γ-mangostin (IC50 = 1.31 µM), 3-isomangostin (IC50 = 5.75 µM), 8-desoxygartanin (IC50 = 20.41 µM), and gartanin (not detected), demonstrating effective AChE inhibition in vitro (Khaw et al. 2014). Thus, these discarded byproducts of mangosteen are worth developing.

## Conclusions

The increasing prevalence of AD and DM necessitates the discovery of new AChE and α-glucosidase inhibitors. This study highlights mangosteen pericarp as a promising natural source for these inhibitors, potentially useful for developing nutraceuticals and pharmaceuticals. Eight potential inhibitors, 8-desoxygartanin, gartanin, 3-isomangostin, β-mangostin, 9-hydroxycalabaxanthone, γ-mangostin, α-mangostin, and garcinone E, were identified from mangosteen pericarp, all of which inhibit both AChE and α-glucosidase. Notably, garcinone E emerged as a new AChE inhibitor candidate. The study validated a systems biology strategy using dereplication and comparative UPLC-ESI-MS/MS metabolomics profiling to detect target-binding molecules in protein-treated and untreated plant extracts. Further confirmation was achieved through molecular docking predictions, mechanism exploration, and compound quantification assays. This method is feasible and time-efficient for screening and identifying enzyme inhibitors or receptor-binding compounds in crude extracts. It can be adapted for other source materials and target enzymes with minor modifications, facilitating rapid screening and characterization of valuable constituents in natural resources for database establishment.

## Electronic supplementary material

Below is the link to the electronic supplementary material.

Supplementary Material 1

## Data Availability

The data used and analyzed for the current study can be obtained from the corresponding author.

## Abbreviations

AD: Alzheimer’s disease
DM: Diabetes mellitus
Ach: Acetylcholine
AChE: Acetylcholinesterase
MPLC: Medium-pressure liquid chromatography
SE: Absorbance of sample experiment
SC: Absorbance of sample control
EC: Absorbance of experimental control
BC: Absorbance of blank control
UPLC-MS: Ultra-performance liquid chromatography -mass spectrometry
I0: Intensity of Blank
IT: Intensity of Test
