# Supplementary material for: Comparative metabolomics of acetylcholinesterase and α-glucosidase inhibitors in pericarp of Garcinia mangostana L
Source: Bot Stud. 2025 May 21;66:13. doi: 10.1186/s40529-025-00460-4 (PMC12095715; doi:10.1186/s40529-025-00460-4)
Supplement: Supplementary file 1 — Supplementary Material 1 [file 40529_2025_460_MOESM1_ESM.docx]

Table S1

| **Protein compounds** | **3A4A (alfa-glucosidase)** | | | | | **1C2B (Acetylcholinesterase)** | | | | |
| --- | --- | --- | --- | --- | --- | --- | --- | --- | --- | --- |
|  | **Binding Energy (△G)** | **cdocker interaction energy** | **H-bond** | **Hydrophobic** | | **Binding Energy (△G)** | **cdocker interaction energy** | **H-bond** | **Hydrophobic** | |
|  |  |  |  | **Pi-Pi Pi-Alkyl** | |  |  |  | **Pi-Pi Pi-Alkyl**  **Stacked/T-shaped** | |
| **ᾳ -mangostin** | -29.54 | -54.6424 | Leu313, Pro312, Glu411, His280 | Tyr158 | Arg315, His280, Val216, Phe303 | \| -21.5838 \| -21.5838 \| \| --- \| --- \| | -43.1375 | Asp74, Tyr72 | Trp286, Tyr124, Tyr341, | Tyr337, Trp86, Leu76 |
| **β-mangostin** | -17.4442 | -48.7201 | Pro312, His280 |  | Arg315, Val216, Phe303, Phe314, Tyr158, Phe178, Lys156 | -19.9443 | -43.9651 | Tyr341 | Trp286, Tyr124, | Tyr337, Trp286, Tyr72, Trp86, Phe338 |
| **γ-mangostin** | -23.9463 | -53.5557 | Leu313, Glu411, His280, Gln279 | Tyr158 | Arg315, Val216, Phe303 | -19.2203 | -43.6012 | Asp74, Tyr72 | Trp286, Tyr124 | Tyr337, Trp86, Leu76, Tyr72 |
| **3-Isomangostin** | -23.8909 | -47.7522 | Pro312, Gln279 |  | Arg315, Phe303, Phe314, Tyr158 Lys156 | -17.3201 | -43.3510 | - | Trp286, Tyr124, Tyr341, | Tyr337, Trp86, Trp286, Tyr124, |
| **8-Desoxygartanin** | -18.1744 | -43.1987 | Asp307 | Phe303 | Arg315, His280, Phe314, Lys156 | -26.3520 | -46.9880 | Arg296, Phe295, Tyr124 | Tyr341, Trp286, | Tyr124, Tyr337, Tyr72, Leu76, |
| **9-hydroxycalabaxanthone** | -20.7818 | -48.0106 | Glu411, His280, Leu313 |  | Arg315, Arg442, Phe303 | -14.0434 | -16.9778 | Asp74, | Tyr124, Trp286, | Trp286, Tyr337 |
| **Gartanin** | -19.0203 | -45.5034 | Asp307  Arg442 | Phe303 | Arg315, Lys156, His280, Tyr158  Phe314 | -24.7447 | -14.003 | Tyr124, Phe295, Arg296, | Tyr341, Trp286, Tyr124 | Leu76, Leu289, Tyr341 |
| **Garcinone_E** | -22.5433 | -55.7964 | Leu313, Pro312, Tyr158, His280 | Tyr158 | Arg315, Phe178, Phe314, Tyr158 Lys156, Val216, His280 | -27.0426 | -47.9602 | - | Trp286, Tyr341,  Tyr124 | Leu289, Arg296, Tyr124 |
| ***Quercetin** | -16.4072 | -41.1975 | Leu313, Pro312  Tyr158, His280,  Glu411, Arg442 | Tyr158 |  |  |  |  |  |  |
| **#** Enserin |  |  |  |  |  | \| -23.1270 \| 14.6927 \| \| --- \| --- \| | -34.2802 | Ser293, Tyr341, Asp74 | Trp286 | Leu289, Trp286, Tyr341, Tyr337 |

*Quercetin as positive for **alfa-glucosidase (PDB:3A4A),** # Enserin as positive for **Acetylcholinesterase (PDB:1C2B).**
